# Understanding the Surface Regeneration and Reactivity of Garnet Solid-State Electrolytes

**DOI:** 10.1021/acsenergylett.3c01042

**Published:** 2023-07-20

**Authors:** Sundeep Vema, Farheen N. Sayed, Supreeth Nagendran, Burcu Karagoz, Christian Sternemann, Michael Paulus, Georg Held, Clare P. Grey

**Affiliations:** † Yusuf Hamied Department of Chemistry, University of Cambridge, Lensfield Road, Cambridge CB2 1EW, United Kingdom; ‡ The Faraday Institution, Quad One, Harwell Campus, Didcot OX11 0RA, United Kingdom; § Diamond Light Source, Harwell Science and Innovation Campus, Didcot OX11 ODE, United Kingdom; ∥ Fakultät Physik/DELTA, Technische Universität Dortmund, 44221 Dortmund, Germany

## Abstract

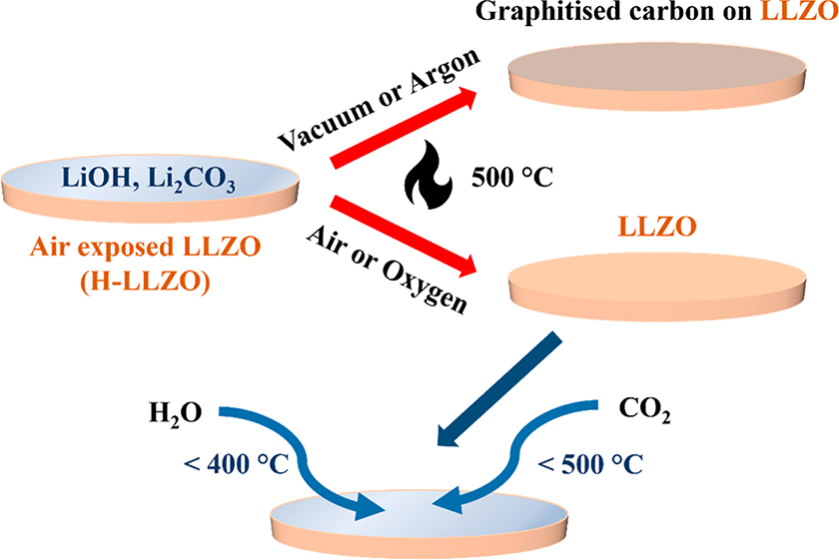

Garnet solid-electrolyte-based Li-metal batteries can be used in energy storage devices with high energy densities and thermal stability. However, the tendency of garnets to form lithium hydroxide and carbonate on the surface in an ambient atmosphere poses significant processing challenges. In this work, the decomposition of surface layers under various gas environments is studied by using two surface-sensitive techniques, near-ambient-pressure X-ray photoelectron spectroscopy and grazing incidence X-ray diffraction. It is found that heating to 500 °C under an oxygen atmosphere (of 1 mbar and above) leads to a clean garnet surface, whereas low oxygen partial pressures (i.e., in argon or vacuum) lead to additional graphitic carbon deposits. The clean surface of garnets reacts directly with moisture and carbon dioxide below 400 and 500 °C, respectively. This suggests that additional CO_2_ concentration controls are needed for the handling of garnets. By heating under O_2_ along with avoiding H_2_O and CO_2_, symmetric cells with less than 10 Ωcm^2^ interface resistance are prepared without the use of any interlayers; plating currents of >1 mA cm^–2^ without dendrite initiation are demonstrated.

Solid-electrolyte (SE)-based Li-metal batteries can enable high-energy storage devices due to their potential compatibility with a Li metal anode and high-voltage cathodes.^1−3^ They offer greater thermal stability than the current state-of-the-art liquid-electrolyte-based Li-ion batteries.^4−6^ Among various SEs explored so far, doped LLZO (Li_7_La_3_Zr_2_O_12_) garnets have high room-temperature (RT) ionic conductivities of 0.1–1 mS cm^–1^ and comparatively wide electrochemical stability, which make them promising candidates for commercial applications.^7−10^

It is well known that LLZO reacts with trace moisture and carbon dioxide in the atmosphere.^11−15^ This results in the exchange of Li^+^ in the lattice with H^+^ to form protonated LLZO (H_*x*_Li_7–*x*_La_3_Zr_2_O_12_) and lithium hydroxide and carbonate surface layers. Protonation leads to lattice contraction and a change of symmetry from *Ia*3̅*d* to *I*4̅3*d*,^16−20^ and the resulting heterogeneous surface layers have very low Li-ion conductivity and thus increase the interfacial resistance when LLZO is, for example, paired with a Li metal anode.^11,12,21,22^ This leads to non-uniform current distribution at the Li–LLZO–Li metal interface, which decreases the critical current density (*I*_CCD_) at which Li metal dendrites nucleate and short-circuit the cell.^13,14,23^

Different protocols for regeneration of LLZO have been reported, wherein the samples have been treated under a variety of gases and temperatures.^21,24−27^ Despite this, the minimum temperature needed to regenerate the surface has not been definitively established, and the effect of different gases has not yet been systematically studied. It has also been reported that excessive heating of LLZO results in pyrochlore formation. This irreversible decomposition has been studied using bulk X-ray diffraction (XRD), and a range of onset temperatures have been reported.^25,28,29^ Since the decomposition starts at the surface of LLZO, careful investigation with surface-sensitive techniques is needed to determine the onset temperature for this reaction accurately. Finally, even though the composition of the surface layers has been characterized, the onset temperature and the reaction mechanisms leading to formation of surface layers are poorly understood; for example, contradicting reports exist on the direct reactivity of LLZO with CO_2_.^11,12,30,31^ The extreme sensitivity of the LLZO surface demands not only surface sensitivity but also *in situ* techniques to understand the regeneration and reactivity of LLZO.

In this study, air-exposed LLZO pellets were heated under different gas environments (vacuum, argon, static air, and flowing air) to study the regeneration process, and *in situ* grazing incidence X-ray diffraction (GIXRD) patterns were collected to capture the structural changes at the surface. To map the chemical composition of the surface, *in situ* near-ambient-pressure X-ray photoelectron spectroscopy (NAP-XPS) spectra were then collected while the samples were heated under vacuum, argon, dry air, and oxygen, with heating under oxygen resulting in complete regeneration and a clean LLZO surface. The samples were then freshly regenerated by heating under oxygen, and *in situ* NAP-XPS spectra were collected while the samples were cooled under H_2_O vapors, CO_2_, and a mixture of H_2_O vapors + CO_2_ to understand the formation of surface layers on LLZO. Finally, by heating air-exposed LLZO samples under oxygen and avoiding CO_2_ and H_2_O during cooling, low Li–LLZO interfacial resistances (<10 Ωcm^2^) were achieved, and dendrite-free plating was obtained at currents above 1 mA cm^–2^.

## GIXRD Measurements under Different Gas Environments

Al-LLZO (with composition Al_0.36_Li_5.92_La_3_Zr_2_O_12_) powder was synthesized using a solid-state method (see methods in the Supporting Information (SI)), hot-pressed, and cut into pellets (∼99% relative density). The phase purity of the pellets was confirmed via synchrotron XRD (Figure S1). GIXRD was then performed in different gas environments. Due to the limitation of the setup, heating could not be performed under pure oxygen, but vacuum (0.01 mbar), argon, static air, and flowing air (1 atm) environments were tested. All samples were first exposed to air for 20 min at RT and then placed into the GIXRD setup in the beamline. A grazing incidence angle of 0.1° was chosen, which corresponds to a probing depth of about ∼3 nm (for LLZO). The samples were heated in controlled gas environments to 800 °C in steps of 100 °C, and GIXRD patterns were collected at each temperature and then on cooling to RT (see experimental details in the SI).

**Figure 1:**
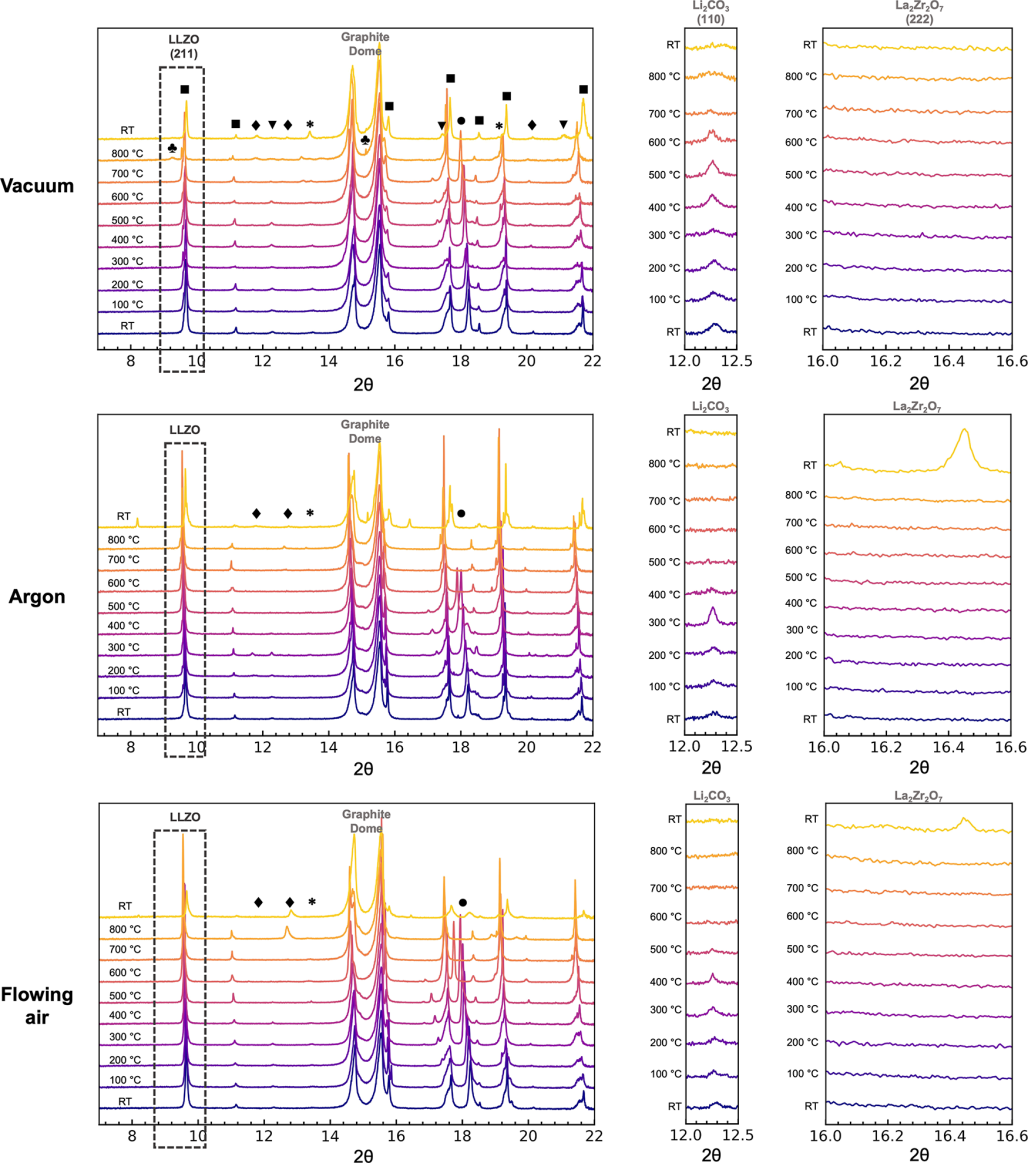
GIXRD (λ = 0.8856 Å) patterns of air-exposed samples heated under different gas environments from RT to 800 °C in 100 °C increments and then cooled to RT. The second and third columns of images are the enlarged versions of the regions corresponding to Li_2_CO_3_ (110) and La_2_Zr_2_O_7_ (222) reflections, respectively. The black dotted-line box and ■ represent LLZO reflections, ⧫ represents Li_2_ZrO_3_, * represents LaAlO_3_, ● represents LiOH, and ▼ represents Li_2_CO_3_. The peaks represented by ★ in the vacuum case at 800 °C could not be indexed to any known compound, and these peaks disappear upon cooling to RT. The shift in the LLZO (211) peaks toward lower 2θ as the sample is heated is due to thermal lattice expansion; sharp discontinuities in the reflections of all the phases are seen between the patterns collected at 800 °C and RT due to rapid lattice contractions.

Lithium carbonate and lithium hydroxide were observed in all air-exposed samples at RT (Figure 1). On heating under vacuum, the Li_2_CO_3_ and LiOH on the LLZO pellets were observed to decompose above 500 °C (reflections marked by ▼ and **●**, respectively, Figure 1, top) and almost completely disappeared by 700 and 800 °C, respectively, in line with previous observations of pure Li_2_CO_3_ decomposition under vacuum.^32^ At 800 °C, formation of Li_2_ZrO_3_ and LaAlO_3_ was observed (reflections marked by ⧫ and *, respectively, Figure 1, top), although no pyrochlore La_2_Zr_2_O_7_ was observed.

Upon heating under argon, Li_2_CO_3_ and LiOH were observed to decompose at the lower temperatures of 400 and 500 °C, respectively. Heating to 800 °C did not result in any observable LLZO decomposition, but the pyrochlore, La_2_Zr_2_O_7_, was detected after the samples were cooled to RT, suggesting LLZO decomposes at elevated temperatures under argon. Under flowing air, Li_2_CO_3_ and LiOH were observed to decompose above 400 and 600 °C, respectively, and La_2_Zr_2_O_7_ pyrochlore was again observed after the samples were cooled to RT as in the argon case, suggesting a similar decomposition mechanism is occurring under flowing air. Interestingly, when samples were heated under static air (under conditions similar to those in a box furnace), Li_2_CO_3_ and LiOH were observed to decompose only above 600 °C, as in the vacuum case (Figure S2). Extensive decomposition of LLZO and pyrochlore formation was also observed upon heating above 500 °C in static air, and the LLZO signal completely disappeared above 600 °C, suggesting that trace moisture in gas environments not removed by gas flow or by pulling a vacuum induces more rapid decomposition of LLZO. We note that Li_2_O evaporation is significantly enhanced by the presence of water (forming the more volatile product, LiOH),^33^ accounting for many of the degradation products seen by GIXRD on heating in static air.

## NAP-XPS Measurements under Different Gas Environments

**Figure 2:**
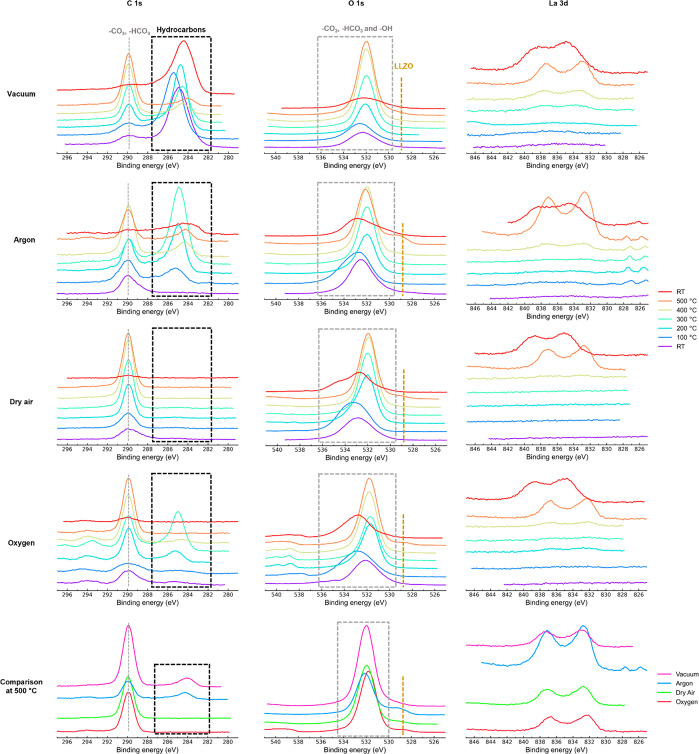
C 1s, O 1s, and La 3d XPS spectra of the air-exposed samples heated under different gas environments from RT to 500 °C in 100 °C increments and then cooled to RT. The gray dotted line and the gray dotted-line box represent Li_2_CO_3_, the golden dotted line represents LLZO, and the black dotted-line box represents surface-adsorbed hydrocarbons and graphitized carbon. The last row compares the C 1s, O 1s, and La 3d XPS spectra at 500 °C under different gas environments.

NAP-XPS was performed to complement the GIXRD observations and map the evolution of the chemical composition of the surface of LLZO pellets during heating. The pellets were polished and stored in a glovebox and were then exposed to air for 20 min before being pumped into the NAP-XPS instrument on the beamline. The incident energy was tuned in a way that the kinetic energy of the ejected photoelectrons probed was ∼200 eV; this corresponds to a probing depth of ∼3 nm for all XPS measurements. The samples were heated in controlled gas (1 mbar, vacuum: 2 × 10^–8^ mbar) environments to 500 °C in steps of 100 °C and then cooled to RT, and XPS spectra were collected during these processes (see experimental details in the SI). The evolution of C 1s, O 1s, and La 3d spectra is presented in Figure 2, while the Li 1s and Zr 3d XPS spectra are shown in Figure S4. The C 1s XPS spectra showed two signals at RT for all samples (Figure 2). Since lithium carbonate is expected to form on the surface of the air-exposed samples, the peak at higher eV was aligned to the Li_2_CO_3_ peak at 289.9 eV.^26^ The XPS spectra of the other elements were shifted by the same amount. Significant asymmetric broadening and shifting of peaks and variations in intensities were observed for all samples due to charging, especially at low temperatures, complicating analysis; thus the 500 °C data are also compared in Figure 2, as they are the most straightforward to analyze.

An additional peak at ∼285 eV was observed in the C 1s spectra in all environments when the samples were heated (Figure 2). This peak disappeared as the samples were heated to 500 °C under dry air and oxygen. By contrast, this peak remained for samples heated under vacuum and argon and stayed even after cooling to RT. This suggests that this peak originates from surface-adsorbed (*sp*^*3*^-containing) hydrocarbons, likely present in the vacuum chamber or in the glovebox, which oxidize in dry air and oxygen but graphitize under low oxygen partial pressures (vacuum and argon), resulting in a shift of this peak to lower eV (284.1 eV; see comparison at 500 °C, Figure 2). Similar observations have been made in previous *in situ* XPS studies performed under vacuum.^25,26^ Our results show that heating under argon can also result in graphitic carbon on the surface of LLZO.

As the samples were heated from RT to 100 °C, the broad O 1s peak shifted toward higher eV. The shift toward higher eV can be attributed to surface -HCO_3_^–^ species^34^ which are enhanced due to surface desorption of H_2_O. Above 100 °C, sharpening of the peak and a shift toward higher eV were observed. The sharpening can be attributed to better charge compensation during XPS measurements at elevated temperatures and the completion of surface-adsorbed water release process. The peak now seen can be assigned to a CO_3_^2–^ species. An additional small peak emerged at 500 °C around ∼529 eV in the O 1s XPS spectra in all gas environments (Figure 2). Sharp peaks were similarly observed in the La 3d and Zr 3d spectra at 500 °C that could not be seen at RT (Figures 2 and S5). Thus, the peak at 529 eV in the O 1s spectra is assigned to the LLZO lattice, confirming the regeneration of LLZO at 500 °C (although the presence of a C 1s Li_2_CO_3_ peak at 500 °C shows incomplete decomposition of the surface layers). The LLZO O 1s peak remains even after cooling to RT (Figure S6) under vacuum, argon, and oxygen but not under dry air.

The GIXRD and XPS results are summarized in Tables S1–S3 and Figure S3. The GIXRD observations suggest that heating until 500 °C can decompose the surface layers on LLZO if the samples are treated under either argon or flowing air (and by extension oxygen). Although the XPS spectra showed incomplete decomposition of the surface layers at 500 °C, this discrepancy might be due to the differences in the way the temperatures of the samples are measured in the two setups. Additional graphitic formation was observed when samples were heated under vacuum and argon. Though recent studies have shown that carbon interlayers reduce Li–LLZO interface resistance and improve performance,^35^ the graphitization on the surface due to heating under argon and vacuum need not be uniform, and any heterogeneity could lead to current focusing when LLZO is used in a SE against Li metal. Finally, avoiding temperatures above 500 °C will prevent pyrochlore formation irrespective of the environments under which samples are heated to regenerate LLZO.

## NAP-XPS Measurements to Study the Onset Temperature for Formation of Surface Layers

**Figure 3:**
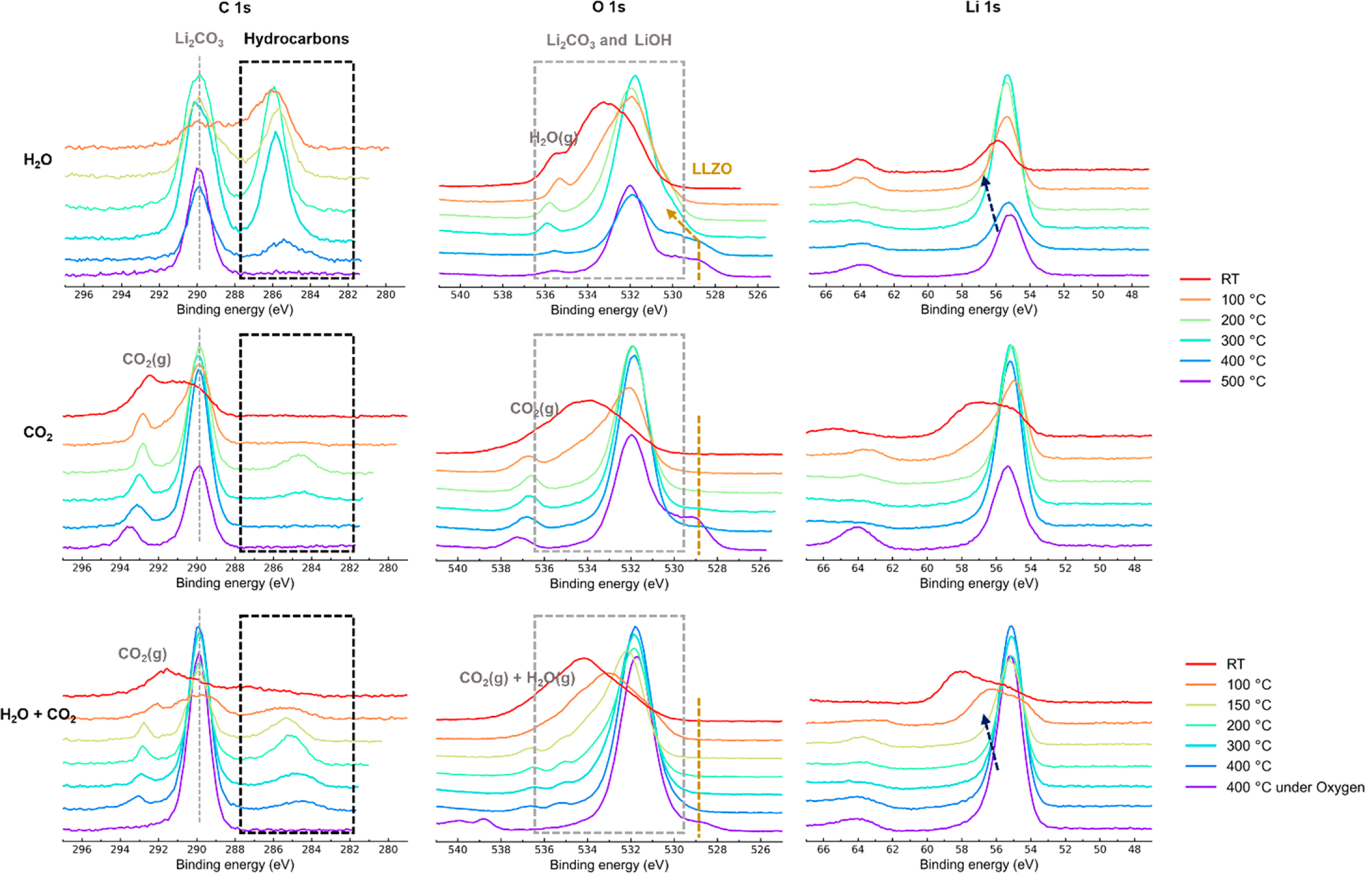
C 1s and O 1s XPS spectra of LLZO during cooling from 500 °C to RT under different gas environments. The gray dotted line represents Li_2_CO_3_, the golden dotted line represents LLZO, the black dotted-line box represents additional hydrocarbon-related species on the surface, and the dark blue dotted line represents LiOH.

To prevent any reformation of surface layers during cooling, it is important to determine the onset temperature for the reaction of LLZO with moisture and CO_2_. LLZO samples were first treated under oxygen at 500 °C for 1 h to allow for surface regeneration, the LLZO lattice peak being observed in O 1s spectra at ∼529 eV, confirming the removal of most of the surface contaminants. Next, H_2_O vapor was introduced into the reaction chamber by manually opening a valve which was connected to a quartz tube containing water (see experimental details in the SI). XPS spectra were then collected while the samples were cooled from 500 °C to RT (Figure 3). The LLZO O 1s lattice peak remained on introduction of H_2_O vapor, and the C 1s spectrum showed a single peak which is attributed to residual Li_2_CO_3_ as discussed above.

As the sample was cooled to 400 °C, an additional peak appeared at around ∼286 eV in the C 1s spectra along with the continued reduction in the intensity of the Li_2_CO_3_ peak. In the O 1s spectra, the Li_2_CO_3_ peak broadened whereas the LLZO lattice peak remained. The additional peak is attributed to oxidized surface-adsorbed hydrocarbons (the shift corresponds to ether-containing hydrocarbons) as observed in the literature when H_2_O is introduced into the XPS chamber.^36^ At 300 °C, the LLZO peak in the O 1s spectra shifted to higher eV, suggesting protonation of LLZO. The Li 1s peak broadened, which is attributed to LiOH formation. The intensity of the additional (∼286 eV) peak in the C 1s spectra increased and was now comparable to that of the Li_2_CO_3_ peak. As the sample was cooled below 200 °C, the LLZO lattice peak completely disappeared, suggesting extensive formation of LiOH on the surface of LLZO, in line with a recent study:^37^
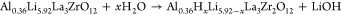
1The additional peak in the C 1s spectra remained even after cooling to RT.

To probe the reaction of carbon dioxide with LLZO, the samples were heated under O_2_ as earlier and CO_2_ was introduced into the reaction chamber (see experimental details in the SI). XPS spectra were collected as the samples were cooled to RT. At 500 °C, the LLZO lattice peak was observed in the O 1s region at ∼529 eV, the peak remaining on introduction of CO_2_. The C 1s spectrum showed a single peak corresponding to Li_2_CO_3_.

Upon cooling to 400 °C, the LLZO peak intensity dropped significantly, suggesting a direct reaction of LLZO with CO_2_. This can be expressed as
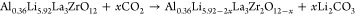
2A reaction of this form requires the extraction of oxygen anions, formally via the extraction of Li_2_O. Thus, this reaction is likely localized just at the surface, as a significant amount of oxygen vacancies need to be generated in the lattice for this reaction to proceed into the bulk of the sample. The reactions with CO_2_ are acid–base reactions, with the basicity of the parent oxide phase Li_2_O (and the Li^+^ mobility) driving the reaction to form Li_2_CO_3_. However, the parent oxide, La_2_O_3_ is more basic, forming La_2_(CO_3_)_3_, which decomposes at high temperatures via the formation of La_2_O_2_CO_3_; La_2_O_2_CO_3_ does not decompose to form La_2_O_3_ until 950 °C under 1 atm of CO_2_.^38^ Thus, reactions of the following form can also occur, written here for the parent LLZO phase to illustrate one possible decomposition pathway:
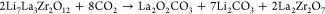
3

While this reaction likely only occurs at the surface, as it involves migration of La^3+^ and Zr^4+^, it does not require the formation of oxygen vacancies in LLZO. It also represents a plausible mechanism in the formation of the pyrochlore phase at higher temperatures (as seen by GIXRD; Table S1), where La^3+^/Zr^4+^ migration can occur. Furthermore, when heated in static air (or any closed vessel), any CO_2_ released from the decomposition of Li_2_CO_3_, which occurs at a lower temperature, can then react to form lanthanum (oxy) carbonate (La_2_O_2_CO_3_).

The presence of any trace water will also result in reactions not requiring the generation of multiple oxygen vacancies:
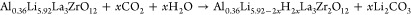
4or
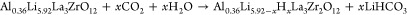
5

As the sample was cooled below 300 °C, the LLZO lattice peak completely disappeared, and a new additional peak was observed in the C 1s spectra around ∼284.8 eV, which can be attributed to surface-adsorbed hydrocarbons. As the sample cooled to 100 °C, this additional peak completely disappeared in the C 1s spectra and a significant broadening of the Li_2_CO_3_ peak was observed. This was accompanied by broadening of the Li_2_CO_3_ O 1s peak, which suggests either extensive reaction of LLZO with CO_2_ or severe charging of the sample. These results also explain the broadening of the O 1s spectra observed at RT after regeneration of LLZO by heating under dry air (Figure S6): the trace amounts of CO_2_ in dry air will react with LLZO below 400 °C and passivate the surface.

In an ambient atmosphere, both H_2_O (∼30 mbar) and CO_2_ (∼0.42 mbar) are present in trace amounts. To check the reactivity of LLZO in this case, LLZO was first heat-treated under O_2_ as in the earlier cases and then cooled to RT under a 1:1 mixture of H_2_O and CO_2_ (see experimental details in the SI). At 400 °C, a reduction and a shift in the LLZO peak toward higher eV were observed, indicating protonation. An additional peak in the C 1s spectra was observed as in the H_2_O and CO_2_ case. Upon further cooling, the Li 1s, C 1s, and O 1s peak evolution was essentially a combination of that seen in the pure H_2_O and pure CO_2_ cases, suggesting reactions described by eqs 3, 4, and 5 are occurring. The summarized results are shown in Table S4.

These results suggest that CO_2_ levels in the environment where LLZO regeneration is performed need to be controlled along with H_2_O levels. This poses a unique challenge for handling LLZO, since CO_2_ levels are not usually monitored and controlled.

Our results should be compared with recent reports in which LLZO/thin-film cathode model systems (with either LiNi_0.6_Mn_0.2_Co_0.2_O_2_ or LiCoO_2_ as the cathode) were sintered in the presence of CO_2_, resulting in enhanced decomposition of both the cathode and LLZO;^39−41^ these results highlight the strong driving force for carbonate formation, even when the LLZO surface is protected via the formation of a LLZO–cathode interface. In contrast, heating to 700 °C under either pure oxygen or an inert atmosphere (N_2_) was shown to result in a low LLZO–cathode interface resistance. A higher interfacial resistance was seen on heating in humidified oxygen at 500 °C, the resistance dropping to a value comparable to the pure O_2_ results, however, on heating to 700 °C. In the current study, by contrast, any moisture was found to result in extensive decomposition of LLZO above 500 °C; the lack of degradation in the presence of moisture in the previous cathode−LLZO systems is ascribed to the protection of the LLZO surface by the cathode film, which helps to reduce LiOH evaporation even in moist environments. In our studies of the bare pellets, the inert atmosphere (argon) led to graphitic deposits on the surface of LLZO, and only oxygen was found to be the ideal gas to regenerate LLZO.

## Electrochemical Studies

**Figure 4:**
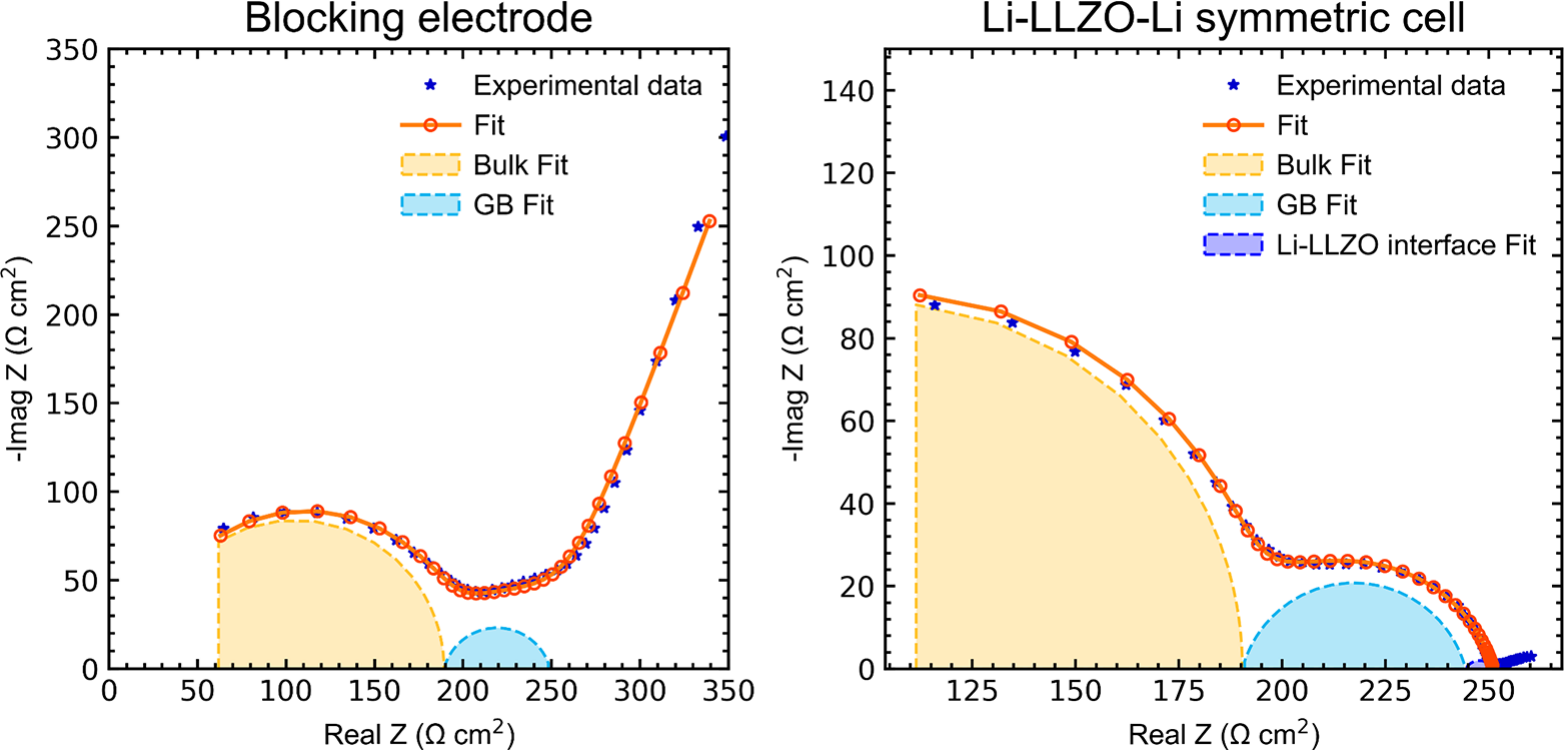
Impedance spectra of (left) a LLZO pellet under blocking conditions, with fits to an equivalent circuit model to determine the bulk and grain boundary contributions, and (right) a Li–LLZO–Li symmetric cell showing the contributions from bulk, grain boundary, and Li–LLZO interface.

Since the NAP-XPS and GIXRD measurements suggested that heating under oxygen (partial pressures of 1 mbar and above) results in the decomposition of surface layers and can lead to complete and clean regeneration of LLZO, the LLZO pellets were treated under oxygen and then transferred to a glovebox without any exposure to air via a custom setup that was purged with argon (Figure S10). Li–LLZO–Li symmetric cells were assembled, impedance measurements were conducted (Figure 4), and the data were fit by using an equivalent circuit model (Figure S7). The Li–LLZO interfacial resistance was found to be <10 Ωcm^2^, in comparison to >500 Ωcm^2^ interfacial resistance shown by samples that had just been polished inside the glovebox (Figure S9). To estimate the *I*_CCD_ before Li dendrites are formed, unidirectional currents were applied to the symmetric cell (see experimental details in the SI). The *I*_CCD_ was found to be >1 mA cm^–2^ (Figure S8), in comparison to the reported *I*_CCD_ of <0.5 mA cm^–2^ for cells with interface resistance >10 Ωcm^2^,^42,43^ suggesting that with an optimized protocol to reduce interface resistance, high plating current densities can be achieved in LLZO garnets.

In conclusion, this work demonstrates that the surface layers (LiOH and Li_2_CO_3_) formed upon exposure of garnets to ambient air can be decomposed by heating to 500 °C, irrespective of the gas environment. Additionally, graphitic carbons have been found to form during the heating of LLZO under vacuum and argon. Though argon is the most common environment for the regeneration of LLZO, heterogeneous graphitization can lead to current focusing and affect the performance of LLZO when used as a SE. Heating above 600–700 °C was found to result in decomposition of LLZO into pyrochlores under argon and flowing air but not under vacuum. A clean LLZO surface was found to react with moisture at temperatures below 400 °C, and direct evidence for reaction of LLZO with CO_2_ was shown below 500 °C. An optimized protocol for regeneration of LLZO is presented, and it is shown that interfacial resistances below 10 Ω·cm^2^ can be achieved for LLZO pellets without the use of any interlayers between Li metal and LLZO. These results mean that the handling of LLZO will require more specialized CO_2_ level controls in addition to the dry rooms used for assembly of liquid-electrolyte-based Li-ion batteries.

## Supporting Information Available

Experimental methods, XRD of samples, additional XPS and GIXRD data, tables summarizing observations from XPS and GIXRD data, equivalent circuit used for fitting impedance and electrochemistry data, and setup used for thermal treatment of samples (PDF)

## References

[ref1] Janek J.; Zeier W. G. A Solid Future for Battery Development. Nat. Energy 2016, 1 (9), 16141 10.1038/nenergy.2016.141.

[ref2] Gao Z.; Sun H.; Fu L.; Ye F.; Zhang Y.; Luo W.; Huang Y. Promises, Challenges, and Recent Progress of Inorganic Solid-State Electrolytes for All-Solid-State Lithium Batteries. Adv. Mater. 2018, 30 (17), 1705702 10.1002/adma.201705702. 29468745

[ref3] Zhang Z.; Shao Y.; Lotsch B.; Hu Y.-S.; Li H.; Janek J.; Nazar L. F.; Nan C.-W.; Maier J.; Armand M.; Chen L. New Horizons for Inorganic Solid State Ion Conductors. Energy Environ. Sci. 2018, 11 (8), 1945–1976. 10.1039/C8EE01053F.

[ref4] Inoue T.; Mukai K. Are All-Solid-State Lithium-Ion Batteries Really Safe?–Verification by Differential Scanning Calorimetry with an All-Inclusive Microcell. ACS Appl. Mater. Interfaces 2017, 9 (2), 1507–1515. 10.1021/acsami.6b13224. 28001045

[ref5] Famprikis T.; Canepa P.; Dawson J. A.; Islam M. S.; Masquelier C. Fundamentals of Inorganic Solid-State Electrolytes for Batteries. Nat. Mater. 2019, 18 (12), 1278–1291. 10.1038/s41563-019-0431-3. 31427742

[ref6] Wu Y.; Wang S.; Li H.; Chen L.; Wu F. Progress in Thermal Stability of All-Solid-State-Li-Ion-Batteries. InfoMat 2021, 3 (8), 827–853. 10.1002/inf2.12224.

[ref7] Zhu Y.; He X.; Mo Y. Origin of Outstanding Stability in the Lithium Solid Electrolyte Materials: Insights from Thermodynamic Analyses Based on First-Principles Calculations. ACS Appl. Mater. Interfaces 2015, 7 (42), 23685–23693. 10.1021/acsami.5b07517. 26440586

[ref8] Thompson T.; Yu S.; Williams L.; Schmidt R. D.; Garcia-Mendez R.; Wolfenstine J.; Allen J. L.; Kioupakis E.; Siegel D. J.; Sakamoto J. Electrochemical Window of the Li-Ion Solid Electrolyte Li_7_La_3_Zr_2_O_12_. ACS Energy Lett. 2017, 2, 462–468. 10.1021/acsenergylett.6b00593.

[ref9] Han F.; Zhu Y.; He X.; Mo Y.; Wang C. Electrochemical Stability of Li_10_GeP_2_S_12_ and Li_7_La_3_Zr_2_O_12_ Solid Electrolytes. Adv. Energy Mater. 2016, 6 (8), 1501590 10.1002/aenm.201501590.

[ref10] Abouali S.; Yim C. H.; Merati A.; Abu-Lebdeh Y.; Thangadurai V. Garnet-Based Solid-State Li Batteries: From Materials Design to Battery Architecture. ACS Energy Lett. 2021, 6, 1920–1941. 10.1021/acsenergylett.1c00401.

[ref11] Cheng L.; Crumlin E. J.; Chen W.; Qiao R.; Hou H.; Franz Lux S.; Zorba V.; Russo R.; Kostecki R.; Liu Z.; Persson K.; Yang W.; Cabana J.; Richardson T.; Chen G.; Doeff M. The Origin of High Electrolyte-Electrode Interfacial Resistances in Lithium Cells Containing Garnet Type Solid Electrolytes. Phys. Chem. Chem. Phys. 2014, 16 (34), 18294–18300. 10.1039/C4CP02921F. 25057850

[ref12] Sharafi A.; Yu S.; Naguib M.; Lee M.; Ma C.; Meyer H. M.; Nanda J.; Chi M.; Siegel D. J.; Sakamoto J. Impact of Air Exposure and Surface Chemistry on Li-Li_7_La_3_Zr_2_O_12_ Interfacial Resistance. J. Mater. Chem. A Mater. 2017, 5 (26), 13475–13487. 10.1039/C7TA03162A.

[ref13] Hofstetter K.; Samson A. J.; Narayanan S.; Thangadurai V. Present Understanding of the Stability of Li-Stuffed Garnets with Moisture, Carbon Dioxide, and Metallic Lithium. J. Power Sources 2018, 390, 297–312. 10.1016/j.jpowsour.2018.04.016.

[ref14] Wang C.; Fu K.; Kammampata S. P.; McOwen D. W.; Samson A. J.; Zhang L.; Hitz G. T.; Nolan A. M.; Wachsman E. D.; Mo Y.; Thangadurai V.; Hu L. Garnet-Type Solid-State Electrolytes: Materials, Interfaces, and Batteries. Chem. Rev. 2020, 120 (10), 4257–4300. 10.1021/acs.chemrev.9b00427. 32271022

[ref15] Huo H.; Luo J.; Thangadurai V.; Guo X.; Nan C. W.; Sun X. Li_2_CO_3_: A Critical Issue for Developing Solid Garnet Batteries. ACS Energy Lett. 2020, 5 (1), 252–262. 10.1021/acsenergylett.9b02401.

[ref16] Galven C.; Suard E.; Mounier D.; Crosnier-Lopez M. P.; le Berre F. Structural Characterization of a New Acentric Protonated Garnet: Li_6-x_H_x_CaLa_2_Nb_2_O_12_. J. Mater. Res. 2013, 28 (16), 2147–2153. 10.1557/jmr.2013.209.

[ref17] Orera A.; Larraz G.; Rodríguez-Velamazán J. A.; Campo J.; Sanjuán M. L. Influence of Li+ and H+ Distribution on the Crystal Structure of Li_7-x_H_x_La_3_Zr_2_O_12_ (0 ≤ x ≤ 5) Garnets. Inorg. Chem. 2016, 55 (3), 1324–1332. 10.1021/acs.inorgchem.5b02708. 26756498

[ref18] Redhammer G. J.; Badami P.; Meven M.; Ganschow S.; Berendts S.; Tippelt G.; Rettenwander D. Wet-Environment-Induced Structural Alterations in Single- And Polycrystalline LLZTO Solid Electrolytes Studied by Diffraction Techniques. ACS Appl. Mater. Interfaces 2021, 13 (1), 350–359. 10.1021/acsami.0c16016. 33372519

[ref19] Redhammer G. J.; Tippelt G.; Portenkirchner A.; Rettenwander D. Aging Behavior of Al- and Ga- Stabilized Li_7_La_3_Zr_2_O_12_ Garnet-Type, Solid-State Electrolyte Based on Powder and Single Crystal X-Ray Diffraction. Crystals (Basel) 2021, 11 (7), 721 10.3390/cryst11070721.

[ref20] Redhammer G. J.; Tippelt G.; Rettenwander D. Deep Hydration of an Li_7–3*x*_La_3_Zr_2_*M*^III^_*x*_O_12_ Solid-State Electrolyte Material: A Case Study on Al- and Ga-Stabilized LLZO. Acta Crystallogr. C Struct Chem. 2022, 78 (1), 1–6. 10.1107/S2053229621012250. 34982043 PMC8725724

[ref21] Sharafi A.; Kazyak E.; Davis A. L.; Yu S.; Thompson T.; Siegel D. J.; Dasgupta N. P.; Sakamoto J. Surface Chemistry Mechanism of Ultra-Low Interfacial Resistance in the Solid-State Electrolyte Li_7_La_3_Zr_2_O_12_. Chem. Mater. 2017, 29 (18), 7961–7968. 10.1021/acs.chemmater.7b03002.

[ref22] Zheng H.; Wu S.; Tian R.; Xu Z.; Zhu H.; Duan H.; Liu H. Intrinsic Lithiophilicity of Li–Garnet Electrolytes Enabling High-Rate Lithium Cycling. Adv. Funct Mater. 2020, 30 (6), 1906189 10.1002/adfm.201906189.

[ref23] Huo H.; Luo J.; Thangadurai V.; Guo X.; Nan C. W.; Sun X. Li_2_CO_3_: A Critical Issue for Developing Solid Garnet Batteries. ACS Energy Lett. 2020, 5 (1), 252–262. 10.1021/acsenergylett.9b02401.

[ref24] Cheng L.; Liu M.; Mehta A.; Xin H.; Lin F.; Persson K.; Chen G.; Crumlin E. J.; Doeff M. Garnet Electrolyte Surface Degradation and Recovery. ACS Appl. Energy Mater. 2018, 1 (12), 7244–7252. 10.1021/acsaem.8b01723.

[ref25] Zhu Y.; Connell J. G.; Tepavcevic S.; Zapol P.; Garcia-Mendez R.; Taylor N. J.; Sakamoto J.; Ingram B. J.; Curtiss L. A.; Freeland J. W.; Fong D. D.; Markovic N. M. Dopant-Dependent Stability of Garnet Solid Electrolyte Interfaces with Lithium Metal. Adv. Energy Mater. 2019, 9 (12), 1803440 10.1002/aenm.201803440.

[ref26] Brugge R. H.; Pesci F. M.; Cavallaro A.; Sole C.; Isaacs M. A.; Kerherve G.; Weatherup R. S.; Aguadero A. The Origin of Chemical Inhomogeneity in Garnet Electrolytes and Its Impact on the Electrochemical Performance. J. Mater. Chem. A Mater. 2020, 8 (28), 14265–14276. 10.1039/D0TA04974C.

[ref27] McConohy G.; Xu X.; Cui T.; Barks E.; Wang S.; Kaeli E.; Melamed C.; Gu X. W.; Chueh W. C. Mechanical Regulation of Lithium Intrusion Probability in Garnet Solid Electrolytes. Nat. Energy 2023, 8 (3), 241–250. 10.1038/s41560-022-01186-4.

[ref28] Grissa R.; Payandeh S.; Heinz M.; Battaglia C. Impact of Protonation on the Electrochemical Performance of Li_7_La_3_Zr_2_O_12_ Garnets. ACS Appl. Mater. Interfaces 2021, 13 (12), 14700–14709. 10.1021/acsami.0c23144. 33729745

[ref29] Cai J.; Polzin B.; Fan L.; Yin L.; Liang Y.; Li X.; Liu Q.; Trask S. E.; Liu Y.; Ren Y.; Meng X.; Chen Z. Stoichiometric Irreversibility of Aged Garnet Electrolytes. Mater. Today Energy 2021, 20, 100669 10.1016/j.mtener.2021.100669.

[ref30] Xia W.; Xu B.; Duan H.; Tang X.; Guo Y.; Kang H.; Li H.; Liu H. Reaction Mechanisms of Lithium Garnet Pellets in Ambient Air: The Effect of Humidity and CO_2_. J. Am. Ceram. Soc. 2017, 100 (7), 2832–2839. 10.1111/jace.14865.

[ref31] Wang Y.; Lai W. Phase Transition in Lithium Garnet Oxide Ionic Conductors Li_7_La_3_Zr_2_O_12_: The Role of Ta Substitution and H_2_O/CO_2_ Exposure. J. Power Sources 2015, 275, 612–620. 10.1016/j.jpowsour.2014.11.062.

[ref32] Dunstan M. T.; Griffin J. M.; Blanc F.; Leskes M.; Grey C. P. Ion Dynamics in Li_2_CO_3_ Studied by Solid-State NMR and First-Principles Calculations. J. Phys. Chem. C 2015, 119 (43), 24255–24264. 10.1021/acs.jpcc.5b06647.

[ref33] Tetenbaum M.; Johnson C. E. Vaporization Behavior of Lithium Oxide: Effect of Water Vapor in Helium Carrier Gas. J. Nucl. Mater. 1984, 120 (2–3), 213–216. 10.1016/0022-3115(84)90058-8.

[ref34] Shchukarev A.; Korolkov D. XPS Study of Group IA Carbonates. Open Chem. 2004, 2 (2), 347–362. 10.2478/BF02475578.

[ref35] Shao Y.; Wang H.; Gong Z.; Wang D.; Zheng B.; Zhu J.; Lu Y.; Hu Y. S.; Guo X.; Li H.; Huang X.; Yang Y.; Nan C. W.; Chen L. Drawing a Soft Interface: An Effective Interfacial Modification Strategy for Garnet-Type Solid-State Li Batteries. ACS Energy Lett. 2018, 3 (6), 1212–1218. 10.1021/acsenergylett.8b00453.

[ref36] Trotochaud L.; Head A. R.; Pletincx S.; Karslloǧlu O.; Yu Y.; Waldner A.; Kyhl L.; Hauffman T.; Terryn H.; Eichhorn B.; Bluhm H. Water Adsorption and Dissociation on Polycrystalline Copper Oxides: Effects of Environmental Contamination and Experimental Protocol. J. Phys. Chem. B 2018, 122 (2), 1000–1008. 10.1021/acs.jpcb.7b10732. 29215283

[ref37] Arinicheva Y.; Guo X.; Gerhards M. T.; Tietz F.; Fattakhova-Rohlfing D.; Finsterbusch M.; Navrotsky A.; Guillon O. Competing Effects in the Hydration Mechanism of a Garnet-Type Li_7_La_3_Zr_2_O_12_ Electrolyte. Chem. Mater. 2022, 34 (4), 1473–1480. 10.1021/acs.chemmater.1c02581.

[ref38] Bakiz B.; Guinneton F.; Arab M.; Benlhachemi A.; Villain S.; Satre P.; Gavarri J.-R. Carbonatation and Decarbonatation Kinetics in the La_2_O_3_ -La_2_O_2_CO_3_ System under CO_2_ Gas Flows. Adv. Mater. Sci. Eng. 2010, 2010, 1–6. 10.1155/2010/360597.

[ref39] Vardar G.; Bowman W. J.; Lu Q.; Wang J.; Chater R. J.; Aguadero A.; Seibert R.; Terry J.; Hunt A.; Waluyo I.; Fong D. D.; Jarry A.; Crumlin E. J.; Hellstrom S. L.; Chiang Y.-M.; Yildiz B. Structure, Chemistry, and Charge Transfer Resistance of the Interface between Li_7_ La_3_ Zr_2_ O_12_ Electrolyte and LiCoO_2_ Cathode. Chem. Mater. 2018, 30 (18), 6259–6276. 10.1021/acs.chemmater.8b01713.

[ref40] Kim Y.; Kim D.; Bliem R.; Vardar G.; Waluyo I.; Hunt A.; Wright J. T.; Katsoudas J. P.; Yildiz B. Thermally Driven Interfacial Degradation between Li_7_La_3_Zr_2_O_12_ Electrolyte and LiNi_0.6_Mn_0.2_Co_0.2_O_2_ Cathode. Chem. Mater. 2020, 32 (22), 9531–9541. 10.1021/acs.chemmater.0c02261.

[ref41] Kim Y.; Waluyo I.; Hunt A.; Yildiz B. Avoiding CO_2_ Improves Thermal Stability at the Interface of Li_7_La_3_Zr_2_O_12_ Electrolyte with Layered Oxide Cathodes. Adv. Energy Mater. 2022, 12 (13), 2102741 10.1002/aenm.202102741.

[ref42] Flatscher F.; Philipp M.; Ganschow S.; Wilkening H. M. R.; Rettenwander D. The Natural Critical Current Density Limit for Li_7_La_3_Zr_2_O_12_ garnets. J. Mater. Chem. A Mater. 2020, 8 (31), 15782–15788. 10.1039/C9TA14177D.

[ref43] Lu Y.; Zhao C.; Yuan H.; Cheng X.; Huang J.; Zhang Q. Critical Current Density in Solid-State Lithium Metal Batteries: Mechanism, Influences, and Strategies. Adv. Funct Mater. 2021, 31 (18), 2009925 10.1002/adfm.202009925.

