# Supplementary material for: Understanding the Surface Regeneration and Reactivity of Garnet Solid-State Electrolytes
Source: ACS Energy Lett. 2023 Jul 20;8(8):3476–84. doi: 10.1021/acsenergylett.3c01042 (PMC10425971; doi:10.1021/acsenergylett.3c01042)
Supplement: Supplementary file 1 — nz3c01042_si_001.pdf [file nz3c01042_si_001.pdf]

Understanding the surface regeneration and reactivity of garnet solid-state electrolytes

Sundeep Vema[1,2], Farheen N. Sayed[1,2], Supreeth Nagendran[1], Burcu Karagoz[3], Christian Sternemann[4], Michael Paulus[4], Georg Held[3], Clare P. Grey[1,*]

[1] Yusuf Hamied Department of Chemistry, University of Cambridge, Cambridge, CB2 1EW, United Kingdom

[2] The Faraday Institution, Quad One, Harwell Campus, Didcot, OX11 0RA, United Kingdom

[3] Diamond Light Source, Harwell Science and Innovation Campus, Didcot, OX11 ODE, United Kingdom

[3] Fakultät Physik/DELTA, Technische Universität Dortmund, Maria-Goeppert-Mayer Straße. 2, 44221 Dortmund, Germany

Supporting Information

Experimental methods

Synthesis of Al-LLZO powder

Al-LLZO was synthesised using a solid-state method. La_2O_3 (99.999%, Alfa Aesar), ZrO_2 (99.9% with 1.8% HfO_2 impurity, Zircomet Cambridge, UK) and Al_2O_3 (TEM <50 nm, Sigma Aldrich) were dried at 900 °C for 12 hours and transferred above 250 °C to a desiccator to allow them to cool down to room temperature (RT). Li_2CO_3 (99.997%, Alfa Aesar) was dried at 150 °C for 12 hours. Precursors corresponding to 30 gm of doped LLZO were stoichiometrically weighed (in the order Zr, Al, La, Li) with 10% excess Li_2CO_3 and then wet milled in one batch with cyclohexane (HPLC grade >99.9%, Alfa Aesar) in Retsch PM100 Ball Mill (~50 mL of 3 mm Ø Zirconia balls, Toray Ceram, Japan in 125 mL Retsch Zirconia jar) at 200 rpm for 5 mins, followed by 100 rpm for 20 hours (10 min active mixing and a 5 min rest cycle). The cap of the jar was opened, and the slurry was mixed thoroughly with a spatula. The mixture was again run at 200 rpm for 1 hour (10 min active mixing and 5 min rest cycle). This protocol was necessary to ensure that the powder did not settle at the bottom of the milling jar and proper mixing of precursors could be achieved. The wet milled slurry was dried over a hotplate at 80 °C and was sieved (150 mm diameter sieve with 1 mm mesh, Glenammer) to separate the Zirconia balls from the dried precursors. The dried powder was filled in MgO

crucibles (SRX61MGO, Almath crucibles) and calcined at 1000 °C for 6 hours under O_2 flow, ~30 mL/min with a 5 °C/min heating rate in a tube furnace (Carbolite) followed by natural cooling. The synthesised doped LLZO powders were transferred above 250 °C to a glovebox to prevent any reaction with moisture and stored in vials.

Induction hot press sintering for Al-LLZO pellets

Al-LLZO powder was first ground in a mortar pestle and sieved using a sieve (100 mm Ø x 75 μm w/w Sieve, 100SBW075, Glenammer). The sieved powder was filled in steps into a custom-made graphite die set (Almath crucibles) and was hot-pressed at 1087 ± 3 °C under 62 MPa pressure using induction coil (IH25A, Across International) and Instron machine under a constant flow of argon. The sample was first heated to 600 °C within ~5 mins and finally to 1085-1090 °C in the next 5 mins and held at this temperature for approximately 50 mins to 75 mins. The samples were cooled once the samples could not be compressed by more than 0.1 mm in the last 20 mins. The samples were cooled to 900 °C in ~ 2 mins, then to 500 °C at the rate of 30 °C per min and followed by natural cooling. The hot-pressed sample was removed from die-set and stored in a desiccator. The phase purity was confirmed using powder X-ray diffraction at the I11 beamline at the diamond light source (λ = 0.824978 Å), Fig. S1.

The hot-pressed samples were stuck on a SS316 cylinder with TriPod wax (41000005, Struers) and cut using a cutting machine (Accutom-50) with a diamond cut-off wheel (102 mm, 4" Ø x 0.3 mm x 12.7 mm Ø, 40000043, Struers) into ~1-1.3 mm thick pellets and 10 mm Ø. The samples were then heated in a box furnace (Carbolite) at 800 °C for 1 hour at the rate of 1 °C/min and then cooled at 10 °C/min to burn off the water-free cutting lubricant (49900070, Struers) used during cutting specimens.

The pellets were first polished using a custom-made setup using 1200 grit size SiC sandpaper (Metprep) to achieve parallel faces. Then the pellets were successively dry-polished to achieve a mirror like finish on a polishing machine, Mecapol (P 255 U, Presi), using 2500 and 4000 grit size SiC sandpaper (Metprep). Both faces of pellets were polished for 4 mins with each sandpaper. Finally, the pellets were wet-polished for 5 mins each side (1 μm diamond suspension, oil-base, 159947 on alpha cloth, 162094, Metprep). Each pellet was immediately cleaned with dry acetone (HPLC grade, Fisher scientific dried with Molecular sieves 3A°, 4 to 8 mesh, Thermo Scientific™, 10104220) and transferred to the glovebox in a vial within minutes after cleaning with dry acetone.

NAP-XPS experiments

NAP-XPS experiments were performed at the B07 beamline, Diamond light source, United Kingdom. Samples stored in glovebox were mounted onto a sample holder under ambient air (which took about 20 mins) and were pumped into the instrument. Depending on the experiment, appropriate gas valves were then opened to maintain a steady pressure in the sample chamber. For heating experiments under argon, oxygen, and dry air and for cooling experiments under CO_2 and H_2O vapours, a pressure of 1 mbar was maintained by tuning the exit valve of the reaction chamber. For experiments under vacuum, the pressure in the experimental chamber was about 2E-08 mbar. H_2O vapours were introduced into the reaction chamber by opening a manually controlled valve which was connected to a quartz tube containing water. CO_2 was introduced into the chamber by opening the valve which was connected to CO_2 gas cylinder. For cooling experiments under a mixture of H_2O vapours and CO_2, the inlet valve of CO_2 was first opened, and the exit valve was tuned to maintain 0.5 mbar pressure, before the H_2O valve was opened. The exit valve was then tuned to maintain a total pressure of 1 mbar.

The x-ray gate was opened, and the incident x-ray energy was set to 1000 eV by choosing the appropriate grating. The sample was then physically aligned to achieve maximum intensity for measurements at every temperature to account for thermal expansion. The pass energy in acquisition mode was set to 40 eV for all measurements. A Survey scan with an incident energy of 1200 eV and a step size of 0.5 eV was performed. Li 1s, Zr 3d, C 1s, O 1s and La 3d XPS spectra were then collected by tuning the incident x-ray energy such that the kinetic energy of the photoelectrons probed was ~ 200 eV. This corresponded to incident energies of 250, 382, 484, 731 and 1036 eV for Li, Zr, C, O and La respectively. These element specific XPS measurements were conducted with a step size of 0.1 eV. Valence band measurements were conducted for each element by changing the kinetic energy of the photoelectrons probed in the acquisition window.

For the heating experiments, the XPS measurements for all elements were first conducted at RT and then the temperature was increased at the rate of 10 °C min^{-1}. The measurements were taken at 100, 200, 300, 400, 500 °C and after cooling down to RT. For the cooling experiments, the samples were first heated under a similar temperature protocol as in the heating experiments under oxygen, before the sample chamber was pumped down to vacuum and then cooled under either CO_2, H_2O, or a 1:1 mixture of H_2O and CO_2 (total pressure of 1 mbar) at the rate of 10

°C min^{-1}. The XPS spectra were collected at an interval of 100 °C. XPS data were then analysed with custom-built Python codes.

GIXRD experiments

GIXRD experiments were performed at the BL9 beamline, DELTA, Centre for Synchrotron radiation, Dortmund, Germany. Samples stored in airtight containers inside the glovebox were opened and exposed to air for 20 mins. The samples were then carefully placed on top of a silver strip inside an Anton Paar DHS 1100 stage setup and a graphite dome was fixed to isolate the sample from ambient atmosphere. The incident x-ray energy was chosen to be 14 keV (0.8856 Å). Next, depending on the experiment, appropriate gas valves were opened to maintain a steady pressure (in case of vacuum: 0.01 mbar) in the sample chamber. The x-ray gate was then opened, and the samples were first aligned with the incident beam to find appropriate zero height and tilt for grazing incidence configuration. Then the incident angle of x-ray beam onto sample was set to 0.1 °. The XRD spectra (2θ from 8 ° to 30 ° in 150 steps with a dwell time of 2s at each step) were then collected at different temperatures. Throughout, the samples had to be aligned at every temperature to account for thermal expansion.

For slow heating experiments under vacuum, argon, static air, and flowing air, the GIXRD measurements were conducted after every 100 °C increment from RT to 800 °C and then after cooling back to RT. The temperature was raised at the rate of 10 °Cmin^{-1} as in the NAP-XPS measurements. For fast heating experiments, the GIXRD measurements were conducted at RT, 500, 600, 700, 800 °C and then after cooling back to RT. GIXRD data were then analysed with home-built Python and MATLAB codes.

Electrochemistry

The polished pellets inside the glovebox were placed in a MgO crucible and transferred to a custom-made quartz tube inside glovebox and thermally etched in a furnace (Carbolite) with a custom-made gas setup to switch between argon and oxygen (Figure S10). The pellets were heated under O_2 at 400 °C for 1 hour then ramped up to 500 °C and heated for 1 hour at the rate of 10 °C/min and then cooled to 200 °C at 10 °C/min. The quartz tube was then purged with argon for 10 mins and then transferred at 200 °C into the glove-box pre-chamber.

Lithium chips (12.7 mm and 0.25 mm thick, PI-KEM) were thoroughly cleaned by placing one lithium chip between two polyethylene plastic films by rubbing the films between fingers to dislodge the surface impurity layers (Oxides, hydroxides, nitrides etc.) to obtain shiny metallic

surface on both sides. The cleaned lithium chip was then rolled between two polyethylene plastic films to reduce its thickness to below 100 μm. Lithium electrodes (6-mm/3-mm diameter) were then punched out of the rolled lithium inside the polyethylene films. The plastic was carefully removed from both sides of the punched-out Li electrode using a Titanium tweezer and the lithium disc was pressed on both sides of the LLZO pellet using a PEEK plastic rod. The pellets with lithium were then heated to 190 °C for 5 mins in Tantalum crucibles (EVCEB-6MMO, Kurt J. Lesker) and then cooled to RT. Next, the pellets were lifted using curved SS316 tweezers and pressed between two punched-out copper foils (12.7 mm diameter). The Li-LLZO-Li sandwich was then heated at 175 °C for a minimum of 30 mins under 1 MPa using a custom-made setup. Next, the pellets were cooled to RT and the copper foils were removed. The electrode area was calculated by image analysis using ImageJ on optical images taken with a Dino-Lite digital microscope camera (AM7115MZTL, GT Vision Ltd.). The samples were then tightly closed in Swagelok cells (SS-810-6BT, Stainless Steel Swagelok Tube Fitting, Bored-Through Union, 1/2 in. Tube OD) for further electrochemical characterization.

For blocking electrode measurements, the thermally etched pellets were centred under two square Kapton masks having a 5 mm Ø hole, and sputter coated with gold on both sides of the pellets. Then the pellets were closed in Swagelok cells for further characterization.

The impedance measurements were performed with an amplitude of 10 mV at frequencies from 7 MHz to 1 Hz using VSP-300 (Biologic). The impedance data was fit with an equivalent circuit using a custom written code in Python to extract the interfacial resistance.

Unidirectional CCD measurements were performed without any external pressure, by successively increasing currents by 0.1 $mAcm^{-2}$, every five minutes until the cell shorted. For checking the long-term stability of plating under the predicted CCD from above experiments, a unidirectional current just below the CCD was applied until the cell reached a cut-off voltage of 5 V.

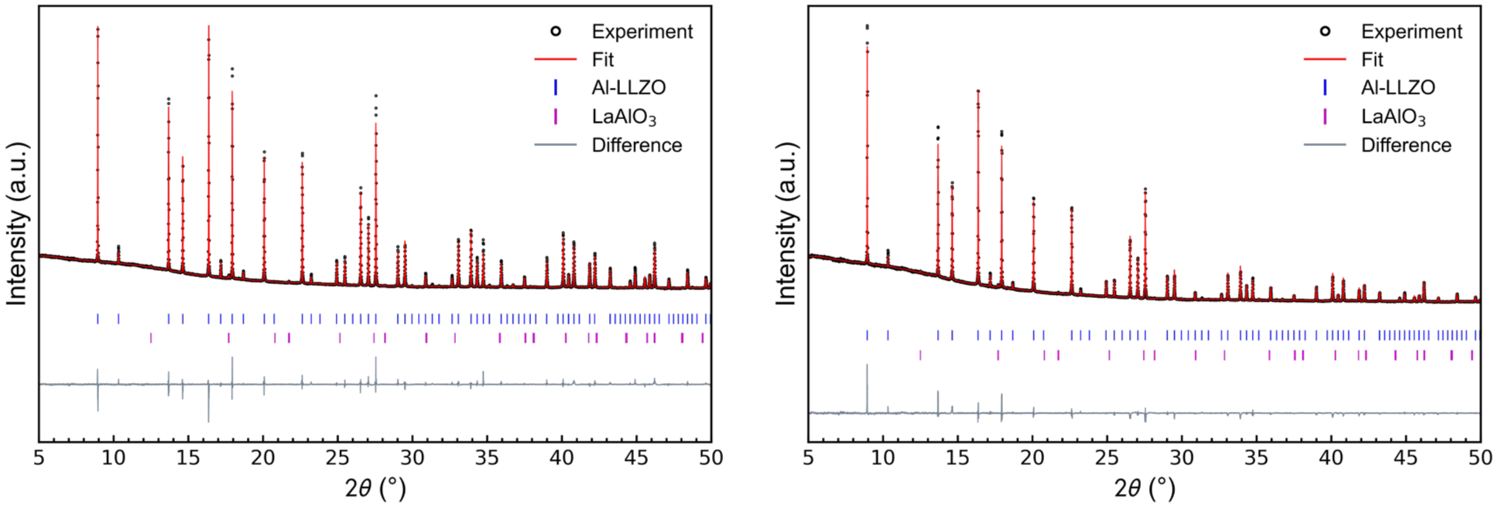

Figure S1: Raw XRD data (black circles) and calculated patterns from Rietveld refinement (red curve) of the Al-LLZO pellets used for NAP-XPS and GIXRD studies (λ = 0.824978 Å). The first set of tick marks (dark blue) indicates the reflections from Ia$\overline{3}$d Al-LLZO phase and the second set (pink) indicates $LaAlO_3$ impurity phase. The grey curve indicates the difference between the fitted and observed data. For this sample the refined lattice parameters were a, b, and c ~ 12.962 Å. The $LaAlO_3$ was ~1.0% in NAP-XPS samples and ~1.7% in GIXRD samples.

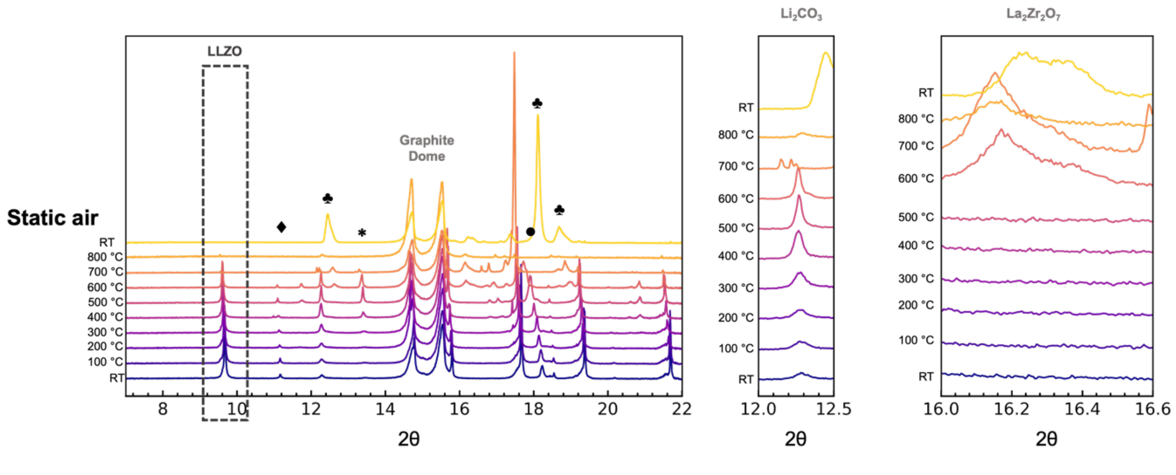

Figure S2: GIXRD (λ = 0.8856 Å) patterns of air exposed samples heated under static air environments from RT to 800 °C in 100 °C increments and then cooled to RT. The second and third column of images are the enlarged versions of the regions corresponding to Li_2CO_3 and $La_2Zr_2O_7$ respectively. The grey dotted-line box contains LLZO reflections, ♦ represents Li_2ZrO_3, ● represents LiOH, and ∗ represents $LaAlO_3$. The shift in LLZO peaks towards lower 2θ as the sample is heated is due to lattice expansion; sharp discontinuities in the reflections of all the phases are seen between the patterns collected at 800 °C and RT due to rapid lattice contractions. The peaks represented by ♣ could not be indexed to any known compound.

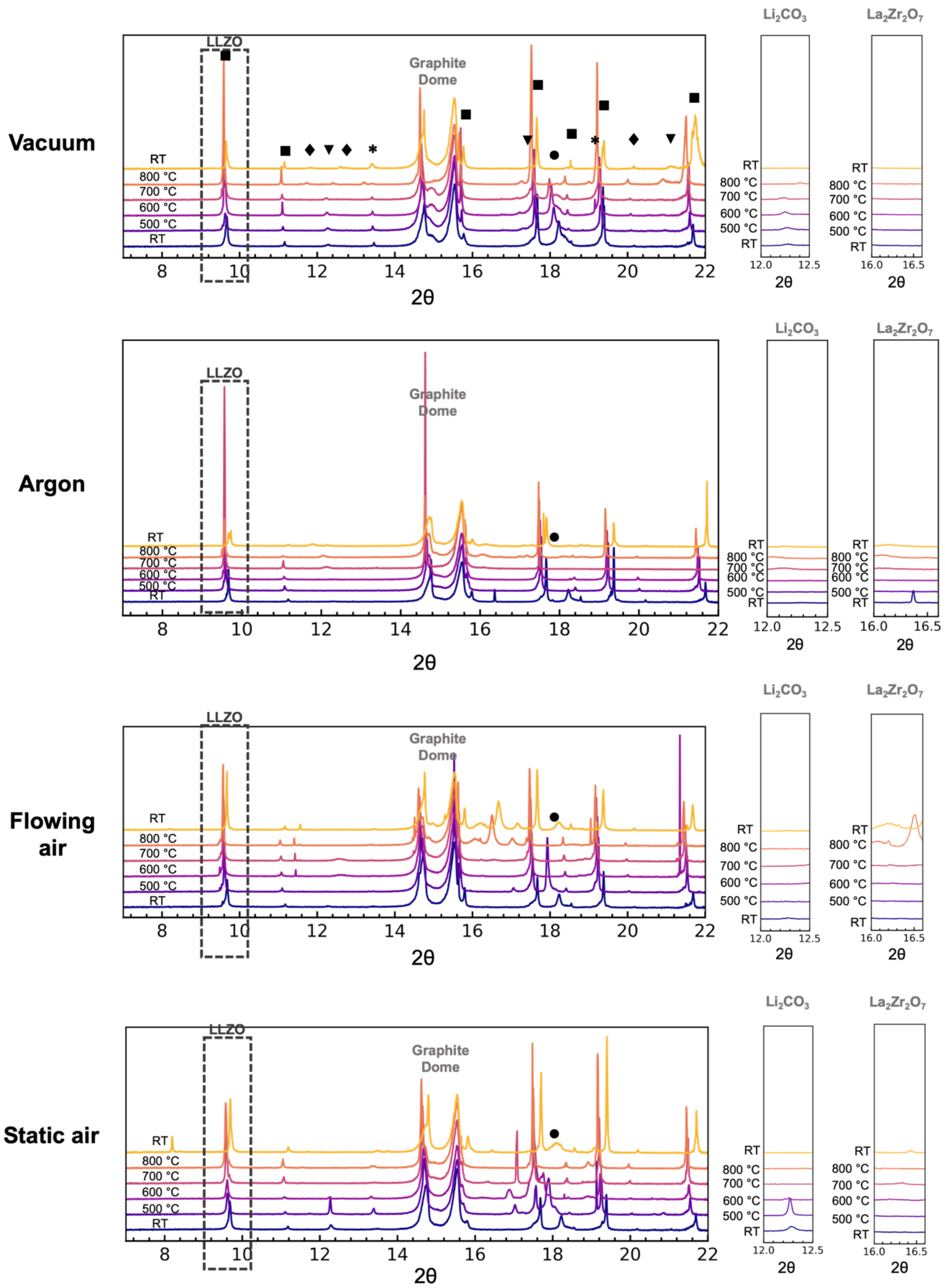

Figure S3: GIXRD (λ = 0.8856 Å) patterns of air exposed samples heated under different gas environments directly from RT to 500°C and then to 800 °C in 100 °C increments and then cooled to RT. The second and third column of images are the enlarged versions of the regions corresponding to Li_2CO_3 and $La_2Zr_2O_7$ respectively. The grey dotted-line box and ■ represents LLZO reflections, ♦ represents Li_2ZrO_3, ∗ represents $LaAlO_3$, ● represents LiOH, and ▼ represents Li_2CO_3. The shift in LLZO peaks towards lower 2θ as the sample is heated is due to

lattice expansion; sharp discontinuities in the reflections of all the phases are seen between the patterns collected at 800 °C and RT due to rapid lattice contractions. Li_2CO_3 was observed to decompose at similar temperatures as in the slow (or step) heating protocol. No pyrochlore formation was observed on heating under vacuum as in the slow (or step) heating protocol whereas it was observed under static air and flowing air. Surprisingly, no pyrochlore was observed under argon unlike in the slow heating protocol suggesting that the decomposition of LLZO under a gas environment is a complex process and requires a further detailed study.

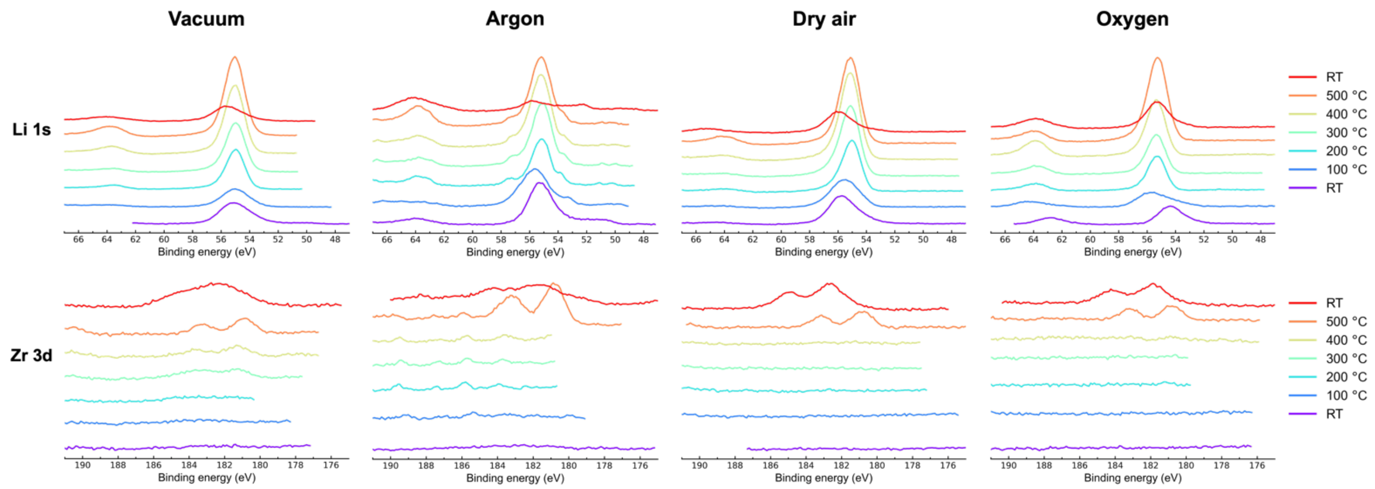

Figure S4: Li 1s and Zr 3d XPS spectra of air exposed samples heated under different gas environments from RT to 500 °C and then cooled to RT

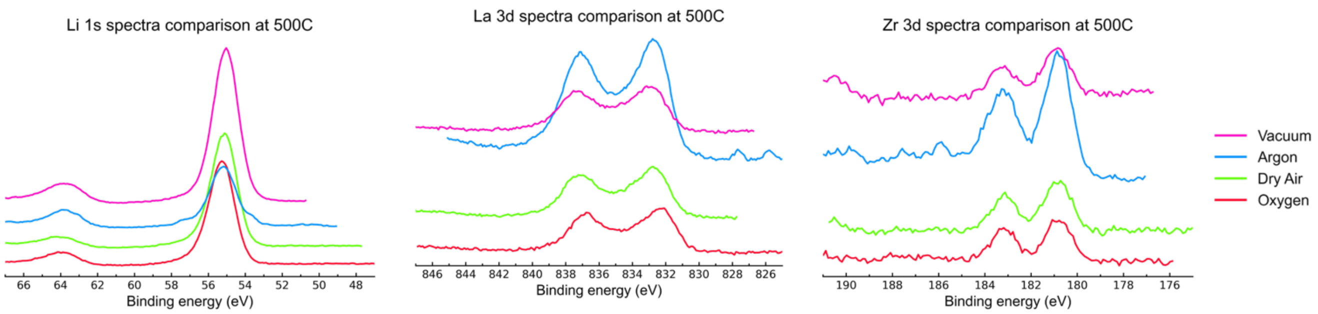

Figure S5: Li 1s, La 3d and Zr 3d XPS spectra comparison at 500 °C after heating under different gas environments

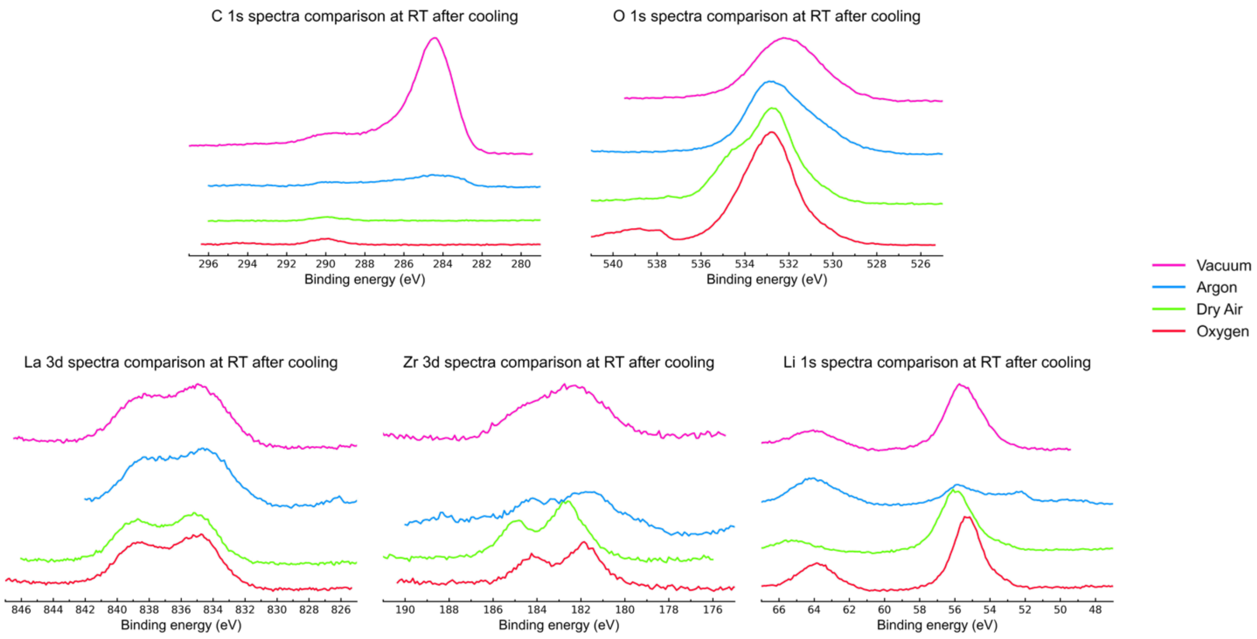

Figure S6: C 1s, O1s, La 3d, Zr 3d and Li 1s XPS spectra at RT after heating under different gas environments

Table S1: Summary of different species observed in GIXRD spectra of samples heated slowly under different gases

It is to be noted that the existence of LiOH above 500 °C (melting point of LiOH is 462 °C) suggests that the temperatures below are not reflective of the exact temperatures experienced by the surface of the sample especially in vacuum. But the relative trends in patterns will still be valid.

<table>
<tr><th>Temperature</th><th>Vacuum</th><th>Argon</th><th>Flowing air</th><th>Static air</th></tr>
<tr><td>RT - 200 °C</td><td colspan="4">LLZO, Li_2CO_3, LiOH and $LaAlO_3$</td></tr>
<tr><td>300 °C</td><td rowspan="4">LLZO, LiOH, Li_2CO_3 and $LaAlO_3$</td><td>LLZO, Li_2CO_3, LiOH, Li_2ZrO_3 and $LaAlO_3$</td><td rowspan="2">LLZO, Li_2CO_3, LiOH and $LaAlO_3$</td><td rowspan="2">LLZO, Li_2CO_3, LiOH and $LaAlO_3$</td></tr>
<tr><td>400 °C</td><td>LLZO, Li_2CO_3, LiOH and $LaAlO_3$</td></tr>
<tr><td>500 °C</td><td>LLZO, LiOH and $LaAlO_3$</td><td rowspan="2">LLZO, LiOH and $LaAlO_3$</td><td>LLZO, Li_2CO_3, LiOH, Li_2ZrO_3 and $LaAlO_3$</td></tr>
<tr><td>600 °C</td><td>LLZO and $LaAlO_3$</td><td>LLZO, Li_2CO_3, LiOH, Li_2ZrO_3, $LaAlO_3$ and $La_2Zr_2O_7$</td></tr>
<tr><td>700 °C</td><td>LLZO, LiOH and $LaAlO_3$</td><td rowspan="2">LLZO, $LaAlO_3$ and Li_2ZrO_3</td><td>LLZO, $LaAlO_3$</td><td>Li_2CO_3, Li_2ZrO_3, $LaAlO_3$ and $La_2Zr_2O_7$</td></tr>
<tr><td>800 °C</td><td rowspan="2">LLZO, $LaAlO_3$ and Li_2ZrO_3</td><td>LLZO, $LaAlO_3$ and Li_2ZrO_3</td><td>Peaks could not be indexed; complex decomposition pathway</td></tr>
<tr><td>RT after cooling</td><td>LLZO, $LaAlO_3$, Li_2ZrO_3 and $La_2Zr_2O_7$</td><td>LLZO, $LaAlO_3$, Li_2ZrO_3 and $La_2Zr_2O_7$</td><td>Peaks could not be indexed; complex decomposition pathway</td></tr>
</table>

Table S2: Summary of origin of different peaks in C 1s and O 1s XPS spectra when samples were heated under different gases

**Temperature**	**Vacuum**	**Argon**	**Dry air**	**Oxygen**
**RT - 200 °C**	Li_2CO_3, LiOH, and hydrocarbons	Li_2CO_3, LiOH, and hydrocarbons	Li_2CO_3, LiOH, and hydrocarbons	Li_2CO_3, LiOH, and hydrocarbons
**300 °C**	Li_2CO_3, LiOH, and partially graphitised hydrocarbons	Li_2CO_3, LiOH, and hydrocarbons	Li_2CO_3, LiOH, and hydrocarbons	Li_2CO_3, LiOH, and hydrocarbons
**400 °C**	Li_2CO_3, LiOH and graphitised carbon	Li_2CO_3, LiOH and graphitised carbon	Li_2CO_3 and LiOH	Li_2CO_3, LiOH and graphitised carbon
**500 °C**	Li_2CO_3, graphitised carbon and LLZO	Li_2CO_3, graphitised carbon and LLZO	Li_2CO_3 and LLZO	Li_2CO_3 and LLZO
**RT after cooling**	Li_2CO_3, graphitised carbon and LLZO	Li_2CO_3, graphitised carbon and LLZO	Li_2CO_3 and LLZO reacted with trace CO_2	Li_2CO_3 and LLZO

To test the effect of heating protocol on the decomposition of the surface layers and the formation of pyrochlore, the samples were heated directly to 500 °C from RT and then to 800 °C in 100 °C increments (Figure S3 and Table S3) while collecting GIXRD spectra (See Experiment details in SI). Similar observations for Li_2CO_3 and LiOH decomposition as in the slow heating case (discussed in main text) could be made suggesting negligible effect of the heating protocol to regenerate the surface of samples which were air exposed for short durations. But the decomposition of LLZO above 500 °C under different gases and heating rates is very complex and requires a further detailed study.

Table S3: Summary of different species observed in GIXRD spectra of samples heated faster under different gases

It is to be noted that the existence of LiOH above 500 °C (melting point of LiOH is 462 °C) suggests that the temperatures below are not reflective of the exact temperatures experienced by the surface of the sample especially in vacuum. But the relative trends in patterns will still be valid.

<table>
<tr><th>Temperature</th><th>Vacuum</th><th>Argon</th><th>Flowing air</th><th>Static air</th></tr>
<tr><td>RT</td><td rowspan="4">LLZO, Li_2CO_3, LiOH and $LaAlO_3$</td><td>LLZO, Li_2CO_3, LiOH, $La_2Zr_2O_7$ and $LaAlO_3$</td><td>LLZO, Li_2CO_3, LiOH and $LaAlO_3$</td><td rowspan="3">LLZO, Li_2CO_3, LiOH and $LaAlO_3$</td></tr>
<tr><td>500 °C</td><td rowspan="2">LLZO</td><td>LLZO, LiOH and $LaAlO_3$</td></tr>
<tr><td>600 °C</td><td>LLZO, $LaAlO_3$ and Li_2ZrO_3</td></tr>
<tr><td>700 °C</td><td>LLZO and $LaAlO_3$</td><td rowspan="2">LLZO, $LaAlO_3$ and Li_2ZrO_3</td><td>LLZO, $LaAlO_3$ and $La_2Zr_2O_7$</td></tr>
<tr><td>800 °C</td><td rowspan="2">LLZO, $LaAlO_3$ and Li_2ZrO_3</td><td>LLZO</td><td>LLZO and $LaAlO_3$</td></tr>
<tr><td>RT after cooling</td><td>LLZO and Li_2ZrO_3</td><td>LLZO, $LaAlO_3$, Li_2ZrO_3 and $La_2Zr_2O_7$</td><td>LLZO, $LaAlO_3$ and $La_2Zr_2O_7$</td></tr>
</table>

Table S4: Summary of origin of different peaks in C 1s, O 1s and Li 1s XPS spectra when samples were cooled under different gases

Temperature	H_2O	CO_2	H_2O + CO_2
**500 °C**	LLZO, Li_2CO_3 and H_2O (g)	LLZO, Li_2CO_3 and CO_2 (g)	N.A.
**400 °C**	LLZO, Li_2CO_3, hydrocarbons, H_2O (g)	Li_2CO_3 and CO_2 (g)	Protonated LLZO, Li_2CO_3, LiOH, hydrocarbons, H_2O (g) and CO_2 (g)
**300 °C**	Protonated LLZO, Li_2CO_3, LiOH, hydrocarbons, H_2O (g)	Li_2CO_3, hydrocarbons and CO_2 (g)	Li_2CO_3, LiOH, hydrocarbons, H_2O (g) and CO_2 (g)
**200 °C**	Protonated LLZO, Li_2CO_3, LiOH, hydrocarbons, H_2O (g)	Li_2CO_3, hydrocarbons and CO_2 (g)	Li_2CO_3, LiOH, hydrocarbons, H_2O (g) and CO_2 (g)
**100 °C**	Protonated LLZO, Li_2CO_3, LiOH, hydrocarbons, H_2O (g)	Li_2CO_3, hydrocarbons and CO_2 (g)	Li_2CO_3, LiOH, hydrocarbons, H_2O (g) and CO_2 (g)
**RT**	Protonated LLZO, Li_2CO_3, LiOH, hydrocarbons, H_2O (g)	Li_2CO_3, hydrocarbons and CO_2 (g)	Li_2CO_3, LiOH, hydrocarbons, H_2O (g) and CO_2 (g)

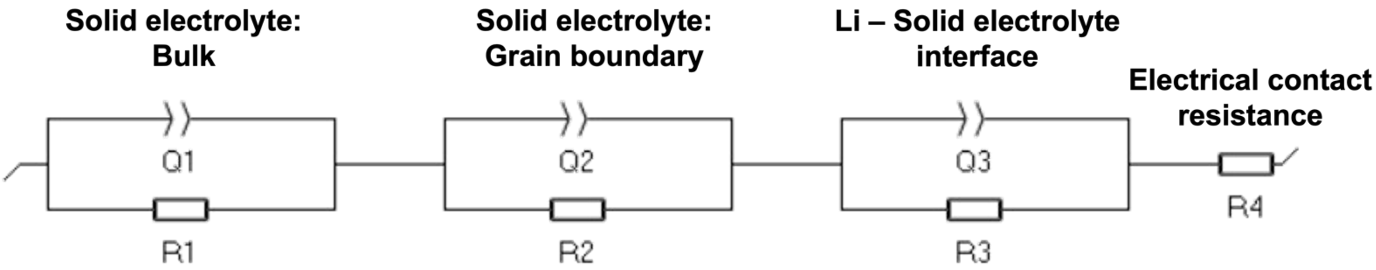

Figure S7: Equivalent circuit for fitting impedance data of symmetric cells, Li-LLZO-Li

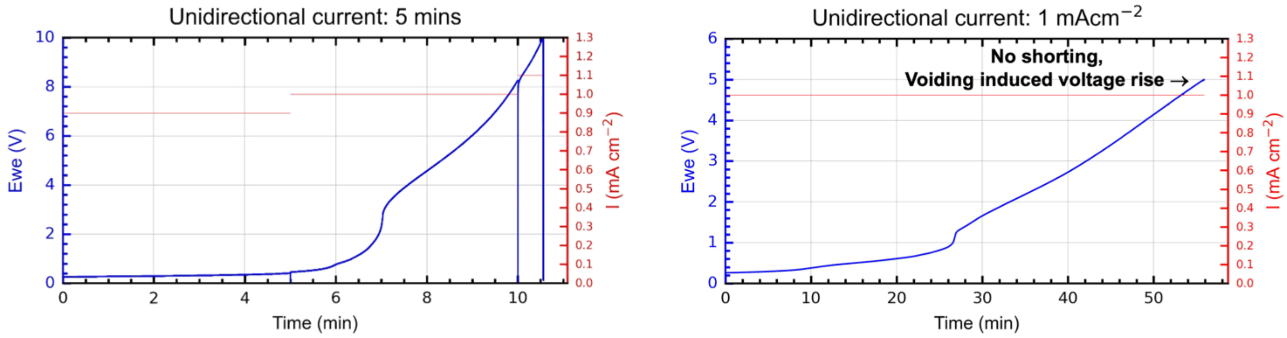

Figure S8: Unidirectional plating experiment with current stepping (+ 0.1 $mAcm^{-2}$) after every 5 mins for estimation of I_{CCD}, long duration plating at 1 $mAcm^{-2}$ without dendrite formation, the cell voltage increases due to void formation on the stripping side

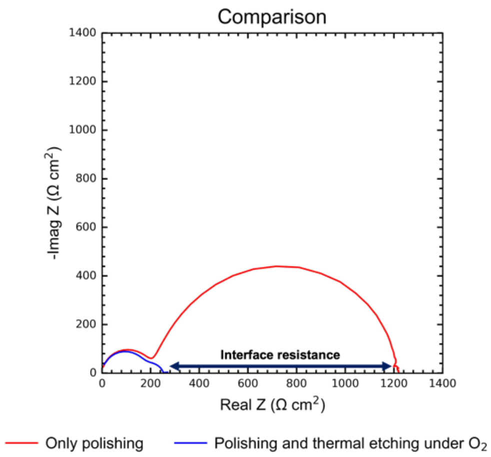

Figure S9: Comparison of interface resistance before and after thermal etching under O_2

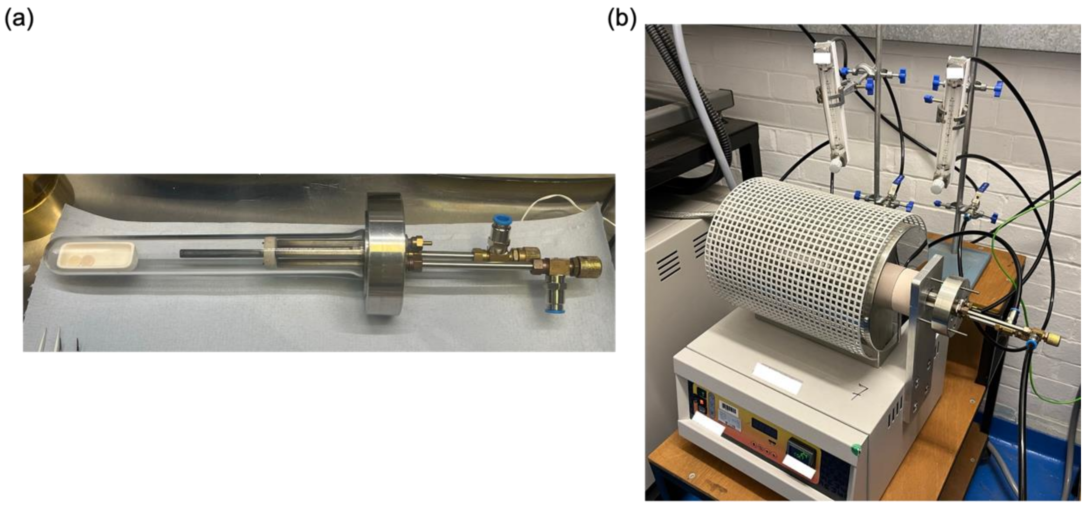

Figure S10: (a) Custom setup to enclose samples in a quartz tube which can be heated under controlled atmosphere without exposure to air and transferred to glovebox without exposure to air. (b) Gas mixing setup
